# A review of the research progress of miR-1237 in malignant tumors

**DOI:** 10.1097/MD.0000000000048065

**Published:** 2026-03-13

**Authors:** Shuyuan Wang, Jingwei Yu, Zhengchao Yan, Weibo Wen, Mengyuan Xin, Xiangdan Li

**Affiliations:** a Department of Morphological Experiment Center, Medical College of Yanbian University, Yanji, Jilin, China.

**Keywords:** malignant tumors, microRNA

## Abstract

MiR-1237, as a type of microRNA, has attracted widespread attention in the field of malignant tumors in recent years. There has been increasing emphasis on its role in regulating tumor occurrence, development, and treatment response. We aim to systematically summarize miR-1237 expression and functional regulation mechanisms in various malignant tumors and explore its potential clinical applications in this review. Our primary objective is to provide a succinct summary of the structural and functional characteristics of miR-1237, as well as elucidate its mechanisms of action within cellular biological processes. Our next step involves meticulously assembling the miR-1237 expression patterns in cancerous tumors and establishing their correlation with clinical and pathological characteristics. We shall lay emphasis on the potential use of this data as a valuable target for tumor prognosis, diagnosis, and therapy. Finally, we will discuss the potential applications of miR-1237 as a tumor therapeutic target in customized care and precision medicine, as well as future approaches for tumor therapy developments. Through an extensive and methodical summary and analysis of the research progress on miR-1237 in malignant tumors, this review seeks to advance the development of precision medicine for tumors, improve the quality of life and survival rate for tumor patients, and encourage the clinical application of miR-1237 in tumor diagnosis, treatment, and prognostic assessment.

## 1. Introduction

The complex regulation of gene expression by miRNAs highlights the importance of these molecules in preserving cellular homeostasis and functionality. Aberrant miRNA expression profiles have been linked to carcinogenesis, metastasis, and resistance to treatment in the setting of cancer, underscoring their potential as prognostic and diagnostic indicators as well as therapeutic targets. MiRNA has a variety of regulatory roles and is evolutionary conserved across species. Its essential function in maintaining homeostasis and development in mammals highlights the intricate interactions between miRNA and its mRNA targets, which control essential biological processes.^[1]^ Furthermore, miRNA displays intricate genomic landscapes among various species.^[2]^ The complexity of physiological processes is reflected in the tissue-specific expression patterns of miRNA. Differences in miRNA expression between species highlight the complex interaction between environmental cues and miRNA.^[3]^ Different miRNA signatures have been found in different tumors by researchers, suggesting that miRNA may be involved in controlling different physiological processes of cancer.^[4]^ MiRNA may play a crucial role in controlling the expression of genes, altering oncogenes and tumor suppressor pathways to aid in the development of tumors.^[5]^ MiRNA precisely regulates cellular behaviors involved in cancer progression, including proliferation, migration, and apoptosis, by fine-tuning key signaling molecules.^[6]^ Further facets of miRNA control are illuminated by endogenous miRNA sponges. These endogenous transcripts, which include circular RNAs (circRNAs), long noncoding RNAs (lncRNAs), and pseudogenes, competitively sequester miRNAs from their target mRNAs, changing patterns of gene expression that are essential for the development and progression of tumors.^[7]^ MiRNA has the potential to be a novel biomarker that targets and regulates the expression of mRNA, hence affecting intricate signal transduction cascades and other biological pathways.^[8]^

In conclusion, the combined discoveries reported in these foundational publications highlight the complex regulatory functions of miRNAs in the biology of cancer. Gaining insight into the molecular processes behind miRNA-mediated gene regulation could greatly aid in the creation of novel therapeutic and diagnostic approaches to treat human malignancies.

## Characteristics of the miRNAs

MicroRNAs (miRNAs) are a class of endogenous RNA molecules approximately 22 nucleotides in length. By cleaving or translationally suppressing target mRNAs, they perform crucial regulatory roles and may have an impact on the expression of several protein-coding genes. Research by David P. Bartel et al^[9]^ has shown that miRNAs regulate gene expression by binding to complementary mRNAs. These miRNA genes have been discovered in diverse animals. Victor Ambros et al.^[10]^ Additionally noted in their research that hundreds of miRNA genes have been found in various animals. Many studies have gradually revealed that the functions of miRNAs are far more extensive than previously recognized, with their regulatory effects being more profound, involving developmental timing, cell death, cell proliferation, hematopoiesis, and neural system construction. The findings from these studies underscore the critical roles of miRNAs in organisms. Research on miRNAs has revealed their significant roles in regulating gene expression, providing new perspectives for understanding gene regulatory networks, and offering potential biomarkers and therapeutic targets for disease diagnosis, treatment, and prognostic assessment.

## Characteristics of the MiR-1237 family

MicroRNAs (miRNAs) are a class of short (20–24 nt) noncoding RNAs found in multicellular organisms that influence translation and mRNA stability to regulate posttranscriptional regulation of gene expression. Of the miRNAs in this class, miR-1237 (MicroRNA-1237) is an RNA gene with unique functions and correlations with diseases. MiR-1237 is a short (20–24 nt) noncoding RNAmiRNA transcribed by RNA polymerase II as part of capped and polyadenylated primary transcripts (pri-miRNAs), which can be protein-coding or noncoding. The primary transcript is cleaved by the Drosha ribonuclease III enzyme, producing approximately 70 nt of stem ring precursor miRNA (precursor miRNA) that is further lysed by cytoplasmic Dicer ribonuclease to produce mature miRNAs and antisense miRNA star (miRNA*) products. Mature miRNAs are incorporated into RNA-induced silencing complexes that recognize target mRNAs by pairing with imperfect bases of the miRNA, most commonly resulting in translational inhibition or instability of the target mRNA. Connection between miR-1237 and afflictions like cysticercosis and spinal cord tumors has been established. If we delve deeper into miR-1237’s expression patterns and functional characteristics in these ailments, we might be able to gain a superior comprehension of its clinical implications and molecular mechanisms. MiR-1237 may be connected to certain illnesses. According to pertinent research, miR-1237 has been connected in several studies to the development and spread of malignant tumors. Variations in its expression levels could have a direct correlation with the molecular pathways involved in malignant tumors.

## 4. Expression of MiR-1237 in cancer

In recent years, comprehensive research has indicated that miR-1237 is closely linked to the initiation and advancement of cancer. The significance of miR-1237’s expression has been emphasized in the cancer-related literature and has aroused the curiosity of researchers, who aspire to delve deeper into its potential role in cancer development.

### 4.1. The role of MiR-1237 in spinal chordoma

Tumors of the spinal cord are uncommon. The most common primary malignant tumors involving the sacrum and mobile spine are called chordomas.^[11]^ Since it is not responsive to radiation or chemotherapy, surgical resection is the primary treatment option; total resection is the ideal surgical technique^[12]^ and postoperative radiation therapy is crucial.^[13]^ The identification of miRNAs may result in novel strategies for spinal chordoma prognosis and treatment.

MiR-1237 has an intimate relationship to the spinal chordoma pathogenesis. After a comprehensive review of 122 relevant articles on spinal chordoma, Wei Huang et al^[14]^ identified 6 miRNAs that had a strong correlation with the survival rate of spinal chordoma patients. One of them that has been shown to have a strong correlation with patient survival is miR-1237-3p. The results showed that the expression of miR-1237-3p was reduced in chordoma tissues and was associated with

Low survival is associated with patients with spinal chordoma. In addition, investigations have shown that miR-1237-3p has the potential to affect the expression of MMP2 and PD-L1, as well as the receptor tyrosine kinase pathway. Consequently, this could have an effect on the biological activity of chordoma cells. This discovery suggests that miR-1237-3p may regulate these signaling pathways and related genes to impact spinal chordoma progression and prognosis. After performing a bioinformatics analysis, it has been determined that miR-1237-3p’s target genes are linked to numerous GO keywords and signaling pathways linked to cancer. This discovery cements its crucial role in the development of spinal chordoma. Additionally, the study has brought to light the substantial regulatory impact of miR-1237 on the EGFR signaling pathway, which plays a vital role in the pathogenesis of spinal chordoma. These results shed light on the molecular mechanisms behind miR-1237 in malignant tumors, particularly with regard to its function in modulating important signaling pathways.

### 4.2. The role of MiR-1237 in gastrointestinal tumors

Malignant tumors of the digestive tract include hepatobiliary tumors, gastric cancer, pancreatic cancer, and colorectal cancer, which is the most prevalent type.^[15]^ In order to improve the prognosis of gastrointestinal malignancies treated with standard therapy, it is important to look for alternative prognostic biomarkers and innovative therapeutics. Among these, immunotherapy is becoming more and more well-known,^[16]^ and mRNA vaccines have great potential in the treatment of gastrointestinal tumors.^[17]^ Therefore, it is necessary to research new miRNA therapeutic targets.

Asahiro Morishita group^[18]^ discovered in colorectal cancer research that blocking miR-1237 can reduce the rate at which colorectal cancer cells proliferate. To study the growth of CW-2 and WiDr cells, researchers transfected them with either negative control miRNAs or miR-1237 inhibitors. It is interesting to note that, 24 hours after transfection with miR-1237 inhibitors, the percentage of surviving cells in CW-2 cells dramatically dropped, but not in WiDr cells. It can be surmised from the observation that the curtailment of miR-1237 brought about by inhibiting Gal-9 compels the decline in the proliferation of colorectal cancer (CRC) cell lineage that exhibits susceptibility towards Gal-9. Furthermore, after receiving Gal-9 therapy, CACO-2 and CW-2 cell lines showed changes in their levels of miRNA expression, and the use of miRNA expression chips allowed researchers to identify miRNAs associated with Gal-9’s antitumor effects. The researchers selected a cohort of microRNAs (miRNAs) that exhibited significant changes in expression levels subsequent to Gal-9 therapy. The differences noted in miRNA expression present a promising opportunity to uncover valuable insights into the molecular mechanisms that contribute to Gal-9’s antitumor properties in individuals with colorectal cancer. The outcomes of the experiment demonstrated that following Gal-9 treatment, miR-1246 was elevated in CACO-2 cells and downregulated in CW-2 cells. Additional research revealed that the deletion of miR-1237 may prevent CW-2 cells from proliferating. Consequently, in Gal-9-sensitive CW-2 cells, Gal-9 suppresses cell growth through miR-1237. This suggests that miR-1237 may be a negative regulatory factor in colorectal cancer, and its inhibition could be a potential therapeutic strategy. Although the primary emphasis of this study is on colorectal cancer, it underscores the prospective therapeutic advantages that miR-1237 may offer in malignant tumors. Additionally, it provides valuable understanding of the mode of operation of this protein in other types of cancer.

Si-Qi Li et al study^[19]^ on plasma expression in colorectal cancer discovered a considerable upregulation of miR-1237 in cancer tissues. This aligns with the downregulation of other miRNAs, leading to the development of a miRNA pentamer that possesses reasonably effective diagnostic abilities with regards to colorectal cancer. These findings suggest that miR-1237 may have tumor-suppressive effects in this particular type of cancer. MicroRNAs (miRNAs) are involved in the genesis and progression of gastric cancer. According to research by Jin-Han Bae et al,^[20]^ miR-1237 is a miRNA that suppresses tumor growth in gastric cancer by silencing DNA hypermethylation. The study conducted by researchers revealed a notable reduction in wound healing, cell migration, and invasion capacity following the transfection of AGS cells with miR-1237 mimics. Significantly, the exogenous production of miR-1237 resulted in a substantial decrease in the expression of proteins linked to the epithelial-mesenchymal transition (EMT), providing support for the concept that miR-1237 hinders tumor growth in gastric cancer.

Additional investigation suggests that miR-1237 may target genes relevant to cancer, including CCND2 and ERbb3. A receptor tyrosine kinase called ERbb3 is intimately linked to the initiation and prognosis of tumors. There is a significant association between tumor growth and a negative prognosis, as well as increased levels of ERBB3, in persons diagnosed with stomach cancer. Conversely, stomach cancer development is significantly influenced by the oncogene CCND2. Tumor cell growth, migration, and invasion can be inhibited by exogenous expression of miR-1237 via blocking the expression of CCND2.

In addition, a variety of biological pathways, including nuclear chromatin, chromatin condensation, and coagulation function, are associated with the target genes of miR-1237. This implies that miR-1237 may control several important biological pathways in order to produce its tumor-suppressive impact.

In conclusion, the production of miR-1237 externally and its subsequent silencing through DNA hypermethylation has shown potential in inhibiting the progression and invasion of stomach cancer. Its target genes include ERbb3 and CCND2, among other cancer-related pathways. Consequently, miR-1237 might be a viable target for stomach cancer treatment in the future.

### 4.3. The role of MiR-1237 in oral cancer

Although there is some degree of preventive measures for oral cancer, the disease has a very high death rate once it is contracted.^[21]^ For this reason, early detection of oral cancer is essential. The most reliable method for identifying oral cancer is a biopsy,^[22]^ and recent studies suggest that identifying miRNA in patients’ bodies may offer a novel approach to the diagnosis of oral cancer.^[23,24]^

In the study of oral squamous cell carcinoma, Christo Rajan team^[25]^ found that the upregulation of miR-1237 is associated with poor prognosis in oral squamous cell carcinoma. The expression of MiR-1237 in oral cancer tissues exhibits a significant upregulation, particularly during the early stages (I and II), and shows a positive correlation with the diminishing tumor depth. This suggests that miR-1237 may play an important role in the occurrence and development of oral cancer, especially in the early stages of tumors.

Further research indicates that miR-1237 is closely associated with the prognosis of oral cancer patients. High expression of miR-1237 is negatively correlated with the survival rate and disease recurrence rate of oral cancer patients, suggesting that it may be an important prognostic marker for oral cancer.

Furthermore, research on the target gene prediction of miR-1237 suggests that it may affect the development of oral cancer by regulating several cancer-associated mechanisms, including transcriptional dysregulation, homologous recombination, endocytosis, and the PI3K-AKT signaling pathway. This provides more evidence for miR-1237’s significant role in oral cancer.

All things considered, miR-1237 is a crucial miRNA that affects the incidence, progression, and prognosis of oral cancer. Its mechanisms of action will become clearer with additional study, which will also offer fresh targets and approaches to the detection and management of oral cancer.

### 4.4. The role of MiR-1237 in nasopharyngeal carcinoma

Nasopharyngeal carcinoma, as a highly prevalent cancer in Southeast Asia, is characterized by poor prognosis and distant metastasis.^[26,27]^ Multiple studies have shown that miRNA plays an important role in nasopharyngeal carcinoma,^[28,29]^ and miRNA is also associated with the recurrence of nasopharyngeal carcinoma.^[30]^ Below, we will discuss the role of miR-1237 in nasopharyngeal carcinoma.

Ting Tang and colleagues conducted research^[31]^ identified AATBC (Apoptosis-Associated Transcript in Bladder Cancer, LOC284837) as a novel lncRNA associated with apoptosis in bladder cancer. AATBC is highly expressed in NPC, and higher levels of AATBC are associated with lower survival rates. AATBC also promotes NPC cell migration and invasion in vitro, and metastasis when in vivo. AATBC increases the expression of the desmosomal PNN protein by sponging microRNA-1237-3p. In turn, PNN interacts with the EMT activator ZEB1, upregulating the expression of ZEB1 and promoting EMT in NPC cells. Together, our results suggest AATBC promotes NPC progression through the miR-1237-3p - PNN - ZEB1 axis.

MiR-1237-3p expression is high in NPC which is associated with poor survival. The research indicates that miR-1237-3p regulates the expression of AATBC (a long noncoding transcript), which is associated with an overexpression. Transwell invasion and wound healing experiments indicate that miR-1237-3p inhibitor transfection promotes migration and invasion of NPC cell, while miR-1237-3p simulant has the opposite effect. These results suggest that miR-1237-3p is responsible for the migration and invasion NPC cells. MiR-1237-3p could be a therapeutic target or a prognostic biomarker due to its important role in regulating AATBC, PNN and ZEB1 axis.

### 4.5. The role of MiR-1237 in renal cancer

The most prevalent type of kidney cancer in children is wilms tumor (WT), which is distinguished by a comparatively low prevalence of related mutations. Two novel methylation regions (DMRs) in WT were discovered by Hanna S. Pereira and her colleagues^[32]^ These DMRs were found in the AURKC and RPS6KA4/MIR-1237 promoters, and both showed significant levels of methylation. All things considered, the researchers’ data support the significance of DNA methylation alterations in WT, underscoring the intricacy of these modifications that impact enhancers, UTRs, gene bodies, and promoter regions in addition to promoter regions.

This study revealed that miR-1237 targets oncogenes, such as D-type cyclin (CCND2) and receptor tyrosine kinase ERBB3 (erb-b2 receptor tyrosine kinase 3), suggesting that miR-1237 functions as a tumor suppressor.

### 4.6. The role of MiR-1237 in gynecological tumors

Studies have demonstrated a strong correlation between the metastasis and recurrence of gynecologic cancer and the EMT. Gynecologic cancer represents a significant concern to women’s health.^[33–35]^ The process of the EMT is also strongly associated with miRNAs.^[36–39]^

Breast cancer can be classified into 5 primary molecular subtypes,^[40,41]^ as well as a few uncommon subtypes. Each of these molecular subtypes has a distinct prognostic impact, and miR-1237 has a different regulatory influence on each of these subtypes. Breast cancer is a common malignant tumor, and HER2-positive (HER2+) breast cancer patients usually have a poor prognosis if they do not respond to targeted therapy. However, the effect of targeted therapy on the endogenous microRNA (miRNA) expression levels is not yet clear. To assess whether miRNAs can make HER2 + cells more sensitive to treatment, Lisa Svartdal Normann et al^[42]^ conducted a high-throughput screening of 1626 miRNA mimics and inhibitors combined with trastuzumab and lapatinib in HER2 + breast cancer cells. The results revealed that 8 miRNA mimics could sensitize cells to targeted therapy, including miR-1237-3p.

Furthermore, cell viability assays showed that these miRNA mimics and inhibitors had minimal impact on cell viability when used alone, but significantly reduced cell viability in the presence of lapatinib and/or trastuzumab. MiR-1237 in breast cancer research interacts with HER2-targeted drugs, significantly affecting cell proliferation. MiR-1237 can increase the sensitivity of KPL4 cells to HER2-targeted drugs. All the evidence gathered from this study indicates that miR-1237-3p, being a potent sensitizing microRNA, could play a valuable role in enhancing the efficacy of therapy for patients dealing with HER2 + breast cancer. These findings provide a potential strategy for combining targeted drugs with miRNAs to improve current HER2 + breast cancer treatment.

MiRNA has been linked to the prognosis and survival rate of ovarian cancer,^[43]^ and it can be utilized as a marker for liquid biopsies of the disease^[43]^ shows that hsa-miR-1237 is closely associated with the survival status of ovarian cancer patients. The study found that hsa-miR-1237 is one of the miRNAs related to survival in ovarian cancer patients. The author also studied the relative expression of has-miR-1237 at different stages in ovarian patients. The expression of has-miR-1237 was not significant in stage I, with fewer cases, while it was more pronounced in stages II, III, and IV. In addition, several studies have discussed the association between miR-1237 and patient prognosis, so we believe that miR-1237 may be expressed during the onset of cancer but becomes significant and persistent in later stages.

One of the most frequent malignant tumors in women is cervical cancer, which is linked to HPV becoming infected,^[44]^ Variations in miRNA expression have been often seen in cervical cancer, indicating that miRNA may be a potential novel biomarker for the disease,^[45]^ suggesting that miRNA can play a role as a new biomarker for cervical cancer. Lastly, Yanru Dong et al^[46]^ revealed the association of hsa-miR-1237 with the progression and prognosis of cervical squamous cell carcinoma (CESC) and endometrial adenocarcinoma. The study suggests that the expression of RILPL2 may be regulated by hsa-miR-1237, with significantly decreased RILPL2 expression observed in cervical cancer tissues. Patients with low RILPL2 expression are more prone to cervical cancer and have a poorer prognosis. Analysis of the TCGA-CESC dataset revealed that the expression of RILPL2 may be influenced by multiple regulatory factors, including DNA methylation and specific miRNAs, among which hsa-miR-1237 was identified as a potential regulatory miRNA for RILPL2. Further investigation into the regulatory mechanism of hsa-miR-1237 on RILPL2 revealed that it may regulate its expression by targeting the 3’UTR region of the RILPL2 gene, leading to mRNA suppression or degradation. This finding provides important clues for understanding the biological function of miR-1237 in cervical cancer. Moreover, hsa-miR-1237 is significantly expressed in CESC tissues, indicating that it controls the expression of RILPL2, which may play a role in the development of cervical cancer. Taken together, hsa-miR-1237 may participate in the occurrence and development of cervical cancer by negatively regulating RILPL2 expression. This new regulatory mechanism opens up new avenues for investigation into the possible uses of miR-1237 in the diagnosis, therapy, and prognosis of cervical cancer. However, further experimental and clinical validation is needed for a detailed understanding of the molecular mechanisms and targets of miRNA-1237 in cervical cancer.

Overall, the variability in the expression levels of miRNA-1237 in different types of cancer may be diverse, but its high expression consistently plays a role in promoting tumor growth and affecting prognosis. We created a table that separately describes the identified factors associated with any specific type of cancer and the corresponding role of miR-1237 (Table 1). Although the exact mechanism of miRNA-1237 in cervical cancer is not yet clear, combining it with research on other types of cancer can provide more clues for further understanding its biological function in cervical cancer. Future studies can delve deeper into the molecular mechanisms of miRNA-1237 in cancer, providing more scientific evidence for its potential application in cancer treatment and prognosis assessment.

**Table 1:** The role of miR-1237 in different cancers and its impact factors.

Cancer	Identified factors	Role of miR-1237
Wilms’ tumor	RPS6KA4	Two novel DMRs located in the RPS6KA4/MIR-1237 and AURKC promoters are hypermethylated in WT.
Lung cancer	COL7A1 overexpression	The silencing properties of miRNAs can be used to design miRNA-1237-mediated therapies that may inhibit overexpressed genes in cancer.
Nasopharyngeal carcinoma	Long noncoding RNA AATBC, desmosome-associated PNN, ZEB1, EMT	AATBC upregulates the expression of desmosome-related PNN through miR-1237-3p sponges. In turn, PNNs interact with the EMT activator ZEB1 and upregulate ZEB1 expression to promote EMT in NPC cells. AATBC promotes nasopharyngeal carcinoma progression via the miR-1237-3p-PNN-ZEB1 axis.
Breast cancer	Trastuzumab and lapatinib are treated	MiR-1237-3p sensitizes HER2 breast cancer cells to targeted therapy.
Colon cancer	Gal-9	miR-1237, significantly altered in vitro and in vivo by Gal-9 treatment, Gal-9-inhibited miR-1237 inhibited cell proliferation in one of the Gal-9-sensitive colon cancer cell lines.

DMRs = differentially methylated regions, EMT = epithelial-mesenchymal transition, MiR = microRNA, PNN = protein pinin, WT = Wilms tumor.

## 5. Conclusion and prospects

Today’s society has increasingly become more interested in customized medicine, and the most promising area of research for this field is miRNA.^[47–49]^ The advancements in the study of miR-1237 in malignant tumors have garnered significant interest, as numerous investigations have demonstrated the protein’s variable expression and modes of action across various cancer kinds. MiR-1237-3p has been found to have a substantial correlation with chordoma patient survival. It may also influence tumor cells’ biological activity via regulating the receptor tyrosine kinase pathway, MMP2 and PD-L1 expression, and other factors. Due to its ability to halt the growth of tumor cells in colon cancer, miR-1237 may prove beneficial as a treatment for the disease. High expression of miR-1237 is linked to a poor prognosis in oral and nasopharyngeal malignancies, and it may influence tumor formation and prognosis through controlling several cancer-related pathways. Additionally, research on miR-1237 in breast and cervical cancers also shows its significant role in tumor growth, invasion, and prognosis.

The field of customized therapy and precision medicine can benefit greatly from the research on miR-1237, which offers crucial details for creating tailored treatment plans. Precision medicine can benefit from its expression patterns and modes of action in various cancer types, which can act as biomarkers to facilitate diagnosis, prognosis evaluation, and treatment plan selection. For instance, the overexpression of miR-1237-3p in HER2-positive breast cancer is linked to susceptibility to targeted therapy, suggesting that it may be used as a supplementary marker for targeted therapy. Moreover, miR-1237 expression is strongly correlated with tumor prognosis, suggesting that it may be used as a prognostic marker to assist identify individuals at high risk and create individualized treatment regimens.

While promising, miR-1237’s application prospects in the field of cancer treatment are not without obstacles. Above all, greater investigation is required to fully understand the mechanisms of action of miR-1237 in various cancer types, particularly with regard to its cross-regulation with signaling pathways pertinent to tumors. Second, in order to assess the expression levels of miR-1237 precisely and provide more solid proof for its clinical use in diagnosis, more accurate detection techniques must be developed. Large-scale clinical trials are also necessary to confirm miR-1237’s clinical usefulness as a therapeutic target and biomarker, advancing the field’s use in tumor precision medicine. It is anticipated that miR-1237 will become more significant in cancer treatment in the future due to the ongoing advancements in technology and in-depth study on miRNAs, which will also improve patient survival and quality of life.

## Author contributions

**Conceptualization:** Shuyuan Wang.

**Funding acquisition:** Xiangdan Li.

**Methodology:** Shuyuan Wang, Jingwei Yu, Zhengchao Yan.

**Supervision:** Xiangdan Li.

**Visualization:** Shuyuan Wang, Weibo Wen.

**Writing – original draft:** Shuyuan Wang.

**Writing – review & editing:** Shuyuan Wang, Mengyuan Xin.
